# Poet, Politician, Exile, and Probable Malaria Victim

**DOI:** 10.3201/eid3007.AC3007

**Published:** 2024-07

**Authors:** Byron Breedlove

**Keywords:** Durante di Alighiero degli Alighieri, Allegorical Portrait of Dante, poet, politician, exile, malaria, Plasmodium, parasites, mosquitoes, vector-borne infections, art and science, About the Cover

**Figure:**
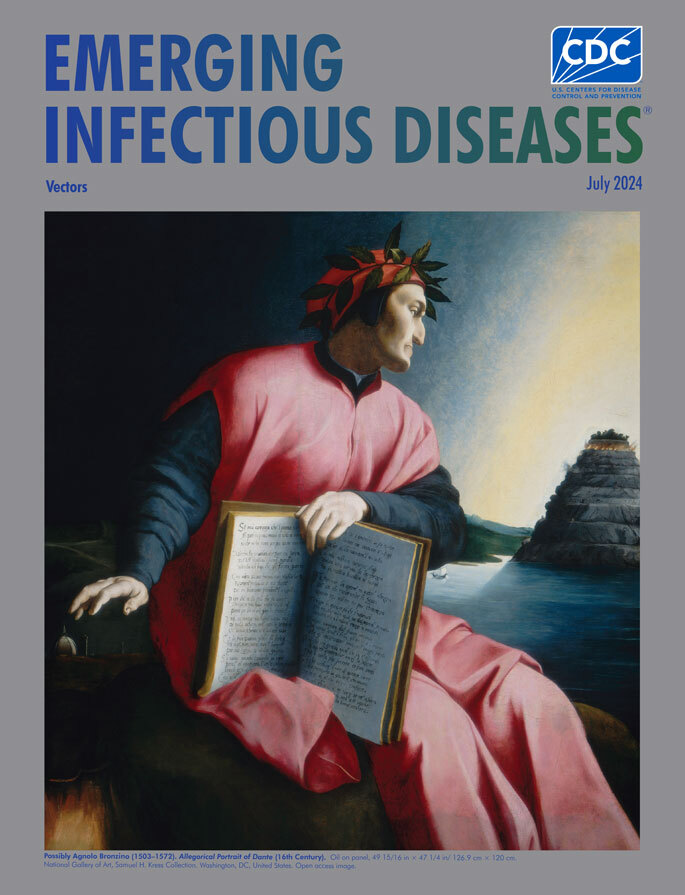
**Possibly Agnolo Bronzino (1503–1572). *Allegorical Portrait of Dante*** (late 16th Century). Oil on panel, 49 15/16 in x 47 1/4 in/126.9 cm x 120 cm. National Gallery of Art, Washington, DC, USA. Open access image.

Poet, politician, and exile all describe Durante di Alighiero degli Alighieri (1265–1321), who is best remembered as the author of *The* *Divine Comedy*. The epic poem chronicles his imagined journey from death to heaven across three realms of the afterlife: *Inferno, Purgatorio,* and *Paradiso*. In 1265, Dante Alighieri was born in Florence, Italy, and details about his early years are scare. He lived in Florence for 35 years, though his political affiliation eventually led to his exile.

During Dante’s lifetime, the Guelphs and the Ghibellines, two rival political factions, vied for control of Florence. The Guelphs backed the papacy; the Ghibellines, the Holy Roman Emperor. Internal squabbles caused the Guelphs to split into the Black Guelphs and the White Guelphs. Professor of Italian Studies Guy P. Raffa explains, “Dante climbed the ladder of Florentine governance as a White Guelph, reaching its highest rung when he was elected to the city’s six-member Council of Priors for a two-month term beginning on June 15, 1300. His triumph could not have come at a worse time. ‘All my woes and all my misfortunes,’ he reflected in a letter, ‘had their cause and origin in my ill-omened election to the priorate.’” Because of trumped-up charges that the Black Guelphs leveled against a number of prominent White Guelphs, Dante was banished from Florence and sentenced to death should he return.

*Allegorical Portrait of Dante*, this month’s cover image, has been credited to different artists. The National Gallery of Art in Washington, DC, which houses the painting, ascribes it to an unknown 16th-century Florentine painter. Others, including art scholar Fiammetta Campagnoli, attribute the portrait to Agnolo Bronzino, a Florentine Mannerist painter. Campagnoli says, “Dante is immediately recognizable from his profile, traditional red clothing and the laurel wreath. Bronzino depicted his subject in front of a landscape background, with a striking turn of the body and as if the viewer is looking up at the figure from below.”

Campagnoli adds that Dante’s right hand “indicates Florence, which is shrouded in darkness, while he himself has turned towards the rising sun that is illuminating the very top of Purgatory and the spheres of Heavenly Paradise.” A small boat visible on the Styx River flows around the tapering tower of Purgatory, and a golden streak from the right corner illuminates the darkened sky. Dante holds *The* *Divine Comedy*, opened to the first 48 lines from Canto XXV of *Paradiso.* Here, as Campagnoli observes, “it is possible to read the lines that speak of the great poet’s desire to return to his homeland”:

then, with another voice and other fleece

a Poet I’ll return, and at the font

of mine own baptism take the laurel crown…

Supported by sympathetic patrons, Dante moved about Italy but never returned to Florence to address corruption charges. Guido Novello da Polenta, lord of Ravenna from 1316 until 1322, was Dante’s final benefactor and provided him, as Raffa explains, “a measure of stability and independence. The poet had his own house in Ravenna, the city in which he found the resources, inspiration, and ambiance conducive to writing the final cantos of the *Divine Comedy*.”

In August 1321, as Ravenna where Dante resided was on the verge of war with the Republic of Venice, Dante was dispatched on a diplomatic mission. Raffa notes, “The land route between Venice and Ravenna posed risks of its own, more so during the time of year when Dante traveled. With the first rains of the season wetting the marshlands, parched after the hot summer months, conditions were ripe for contracting malaria. The rivers, canals, swamps, and lagunas of the region have always made it a fertile haven for mosquito-borne illnesses. By the time Dante returned to Ravenna in early September, the recurring bouts of fever had so weakened him that he died within days.” It is plausible that the fevers Dante experienced were caused by malaria contracted during his travels—especially given the short incubation period (weeks) for malaria. Raffa added, “Although early chroniclers and biographers say precious little about Dante’s final days, their accounts, supplemented by contextual documentation, allow for a plausible representation of his illness, death, and burial.”

Pathologist Luigi Papi offers a similar perspective: “Concerning Dante Alighieri, however, it should be highlighted that there is no historical document which confirms the fact that his death was actually due to malaria, so this hypothesis is probably based only on the fact that at any rate, he died a few days after his long journey by land, through the marshes of the Venetian lagoon where the *Plasmodium* was particularly widespread.” According to the World Health Organization, malaria is caused by 5 species of the *Plasmodium* parasites that Papi refers to, notably *Plasmodium falciparum* and *Plasmodium vivax,* and the vectors for transmission are infected *Anopheles* mosquitoes.

Dante was aware of malaria (which means “bad air” in Italian and comes from *malum aeris* in Latin). Paleopathologist Rafaella Bianucci et al. observe that Dante refers to malaria in several passages of the *Inferno*, including lines 85–90 from Canto XVII:

Like one with quartan-fever’s chill so near,

that pale already are his fingernails, and that, but looking at the shade, he shudders;

such at the words he uttered I became;

but that shame made its threats to me, which renders,

a servant strong when in a good lord’s presence.

At this point in the poem, Dante has mounted Geryon— often described as a mythological monster with three heads—that will transport him and Virgil to the next level of hell. Bianucci et al. note that “Dante is afraid and compares himself to a man in a shivering fit of quartan fever. Dante expresses himself using terms that are very close to those used in medical treatises to exemplify the quartan fever, which reoccurs at regular intervals, oppresses the sick in the fear of the recurring attack.”

More than 700 years after Dante’s death, malaria is preventable and treatable yet kills more people than any other vectorborne disease. Despite gains in controlling malaria in *Plasmodium*-endemic regions, progress is hindered by interlinked factors, including international travel and migration, climate change, and antimalarial drug resistance. Improved surveillance, detection, prevention, and treatment may yield promise not just for controlling outbreaks of malaria but also for dealing with outbreaks of other vectorborne illnesses such as dengue, Crimean-Congo hemorrhagic fever, chikungunya, yellow fever, and Zika, which have also killed thousands of people and taxed systems since the start of this century.
